# Adaptive Extreme Edge Computing for Wearable Devices

**DOI:** 10.3389/fnins.2021.611300

**Published:** 2021-05-11

**Authors:** Erika Covi, Elisa Donati, Xiangpeng Liang, David Kappel, Hadi Heidari, Melika Payvand, Wei Wang

**Affiliations:** ^1^NaMLab gGmbH, Dresden, Germany; ^2^Institute of Neuroinformatics, University of Zurich, Eidgenössische Technische Hochschule Zürich (ETHZ), Zurich, Switzerland; ^3^Microelectronics Lab, James Watt School of Engineering, University of Glasgow, Glasgow, United Kingdom; ^4^Bernstein Center for Computational Neuroscience, III Physikalisches Institut–Biophysik, Georg-August Universität, Göttingen, Germany; ^5^The Andrew and Erna Viterbi Department of Electrical Engineering, Technion–Israel Institute of Technology, Haifa, Israel

**Keywords:** neuromorphic computing, edge computing, wearable devices, learning algorithms, memristive devices

## Abstract

Wearable devices are a fast-growing technology with impact on personal healthcare for both society and economy. Due to the widespread of sensors in pervasive and distributed networks, power consumption, processing speed, and system adaptation are vital in future smart wearable devices. The visioning and forecasting of how to bring computation to the edge in smart sensors have already begun, with an aspiration to provide adaptive extreme edge computing. Here, we provide a holistic view of hardware and theoretical solutions toward smart wearable devices that can provide guidance to research in this pervasive computing era. We propose various solutions for biologically plausible models for continual learning in neuromorphic computing technologies for wearable sensors. To envision this concept, we provide a systematic outline in which prospective low power and low latency scenarios of wearable sensors in neuromorphic platforms are expected. We successively describe vital potential landscapes of neuromorphic processors exploiting complementary metal-oxide semiconductors (CMOS) and emerging memory technologies (e.g., memristive devices). Furthermore, we evaluate the requirements for edge computing within wearable devices in terms of footprint, power consumption, latency, and data size. We additionally investigate the challenges beyond neuromorphic computing hardware, algorithms and devices that could impede enhancement of adaptive edge computing in smart wearable devices.

## 1. Introduction

Wearable devices can monitor various human body symptoms ranging from heart, respiration, movement, to brain activities. Such miniaturized devices using different sensors can detect, predict, and analyze the physical performance, physiological status, biochemical composition, and mental alertness of the human body. Despite advances in novel materials that can improve the resolution and sensitivity of sensors, modern wearable devices are facing various challenges, such as low computing capability, high power consumption, high amount of data to be transmitted, and low speed of the data transmission. Conventional wearable sensing solutions mostly transmit the collected data to external servers for off-chip computing and processing. This approach typically creates an information bottleneck acting as one of the major limiting factors in lowering the power consumption and improving the speed of the operation of the sensing systems. In addition, the use of conventional remote servers with conventional signal processing techniques for processing these temporal real-time sensing data makes it computationally intensive and results in significant power consumption and hardware occupation. In this scenario, the edge computing paradigm, whose definition typically includes all the networks where the computation node is not in the cloud, has become very attractive. Indeed, the closer the computing unit to the sensing one, the more power efficient. In particular, a system is defined able of “extreme edge computing” when the data processing occurs right next to the sensor, on the same device (Rubino et al., 2021). This paradigm calls for a radical shift of perspective. Indeed, general-purpose systems are powerful and versatile, but they do not take the diversity of the quantity and quality of the information generated by different devices into account. In this respect, a custom solution which optimizes the available resources to perform the task at hand might prove to be more advantageous in terms of power, area, and latency than a general-purpose one. Moreover, even when computing is moved to the extreme edge, standard processing units might not provide the ideal solution to the aforementioned issues. Standard von-Neumann architectures feature a physical separation between memory and processing unit, thus further increasing the power consumption to shuttle data between units. Such solutions always need a trade-off between power lifetime and computing capability. Bringing computing at the edge enables faster response times and opens the possibility of personalized always-on wearable devices able for continuously interacting and learning with the environment. However, a radical change of paradigm which uses innovative algorithms, circuits and memory devices is needed to maximize the system performance whilst keeping power and memory budgets at a minimum.

Conventional computers, using Boolean and bit-precise digital representations and executing operations with time-multiplexed and clocked signal, are not optimized for fuzzy inputs and complex cognitive tasks, such as pattern recognition, time series prediction, and decision making. Deep ANNs on the other hand have demonstrated amazing results in a wide range of pattern recognition tasks including machine vision, Natural Language Processing (NLP), and speech recognition (LeCun et al., 2015; Schmidhuber, 2015). Dedicated hardware Artificial Neural Network (ANN) accelerators, including GPUs, TPUs, and custom ASICs with parallel architectures are being developed to execute these algorithms and obtain high accuracy inference results. GPUs provide a substrate well-suited to the parallel processing nature of the ANNs and their very long memory bus is particularly apt for running VMMs, which are at the core of the processing in deep neural networks. Therefore, GPUs support the parallelism, though still pales in comparison to the scale of parallelism that exists in the brain, but they consume orders of magnitude more power than that of the brain (Silver et al., 2016), since they are clocked and the memory access is not localized. To solve this problem, Application Specific Integrated Circuit (ASIC) accelerators try to reduce the complexity of the structure by making the system more application specific and using clock gating and specific hardware structure which matches best to the structure of the mapped neural network to reduce power consumption through less memory read and data access (Cavigelli and Benini, 2016; Chen et al., 2016; Lee et al., 2019; Song et al., 2019). For a complete survey on the state-of-the-art ASIC accelerators for biomedical signals refer to Azghadi et al. (2020).

To go even further in power savings, there are two problems to be solved: (i) remove clock and (ii) perform computation with co-localization of memory and processor. The first problem calls for the development of event-based systems, where processing is performed “asynchronously,” i.e., only when there are input “events.” The algorithmic basis for this kind of “asynchronous” processing is Spiking Neural Network (SNN), in which neurons spike asynchronously only to communicate information to each other.

To avoid the data movement between the memory and the processor, the memory element should be not only used to store data but also to perform computation inside the processor. This approach is called “in-memory computing.” These two approaches of (i) event-based systems and (ii) in-memory computing, together with (iii) massive parallelism, are the three fundamental principles which have led to the development of neuromorphic computing, and to the realization of highly efficient neuromorphic platforms (Schemmel et al., 2010; Furber et al., 2014; Merolla et al., 2014; Moradi et al., 2017; Davies et al., 2018; Frenkel et al., 2019a). Therefore, in this article, we will refer to event-based highly parallel systems that are able to perform real-time sensory processing.

Despite that current fully Complementary Metal-Oxide-Semiconductor (CMOS) implementations of neuromorphic platforms have shown remarkable performance in terms of power efficiency and classification accuracy, there are still some bottlenecks hindering the design of embedded sensing and processing systems. First, the memory used is typically Static Random Access Memory (SRAM), which has very low static power consumption, but it is a large element (six transistors per cell) and it is volatile. The latter feature implies that the information about the network configuration has to be stored elsewhere and transferred to the system at its startup. For large networks, it may take tens of minutes before the system is ready for normal operation. Second, always-on adaptive systems need to work with time constants that have the same time-span of the task that is being learned (e.g., longer than seconds). Implementing such long time constants in neuromorphic CMOS circuits is impractical, since it requires large area capacitors.

To overcome the limitations of fully CMOS-based approaches, the intrinsic unique physical properties of emerging memristive devices can be exploited for both long-term (non-volatile) weight storage and short-term (volatile) task-relevant timescales. In particular, non-volatile devices feature retention times on a long time scale (>10 years, Cheng et al., 2012; Udayakumar et al., 2013; Goux et al., 2014; Golonzka et al., 2018) while showing weight reconfigurability with voltages compatible with typical CMOS circuits (≤3.3 V). Volatile devices, instead, can have time constants on the order of tens of milliseconds to seconds (Jo et al., 2015; Wang et al., 2017; Wang et al., 2019; Wang et al., 2019c; Yang et al., 2017; Covi et al., 2019), thus being able to emulate biological time constants. This feature is especially useful to implement spatiotemporal recognition (Wang et al., 2021) or to enable brain inspired algorithms which need to keep trace of the recent neural activity. This non-volatile/volatile property of memristive devices, together with a small footprint and power efficiency, has indeed attracted a lot of interest in the last 10 years (Linares-Barranco and Serrano-Gotarredona, 2009; Ielmini and Wong, 2018; Chicca and Indiveri, 2020). However, memristive technology has to be supported by *ad hoc* theoretically sound biologically plausible algorithms enabling continual learning and capable to exploit the intrinsic physical properties of memristive devices, such as stochasticity, to achieve accuracy performance comparable to state-of-the-art ANN whilst reducing the power consumption.

**Figure 1:**
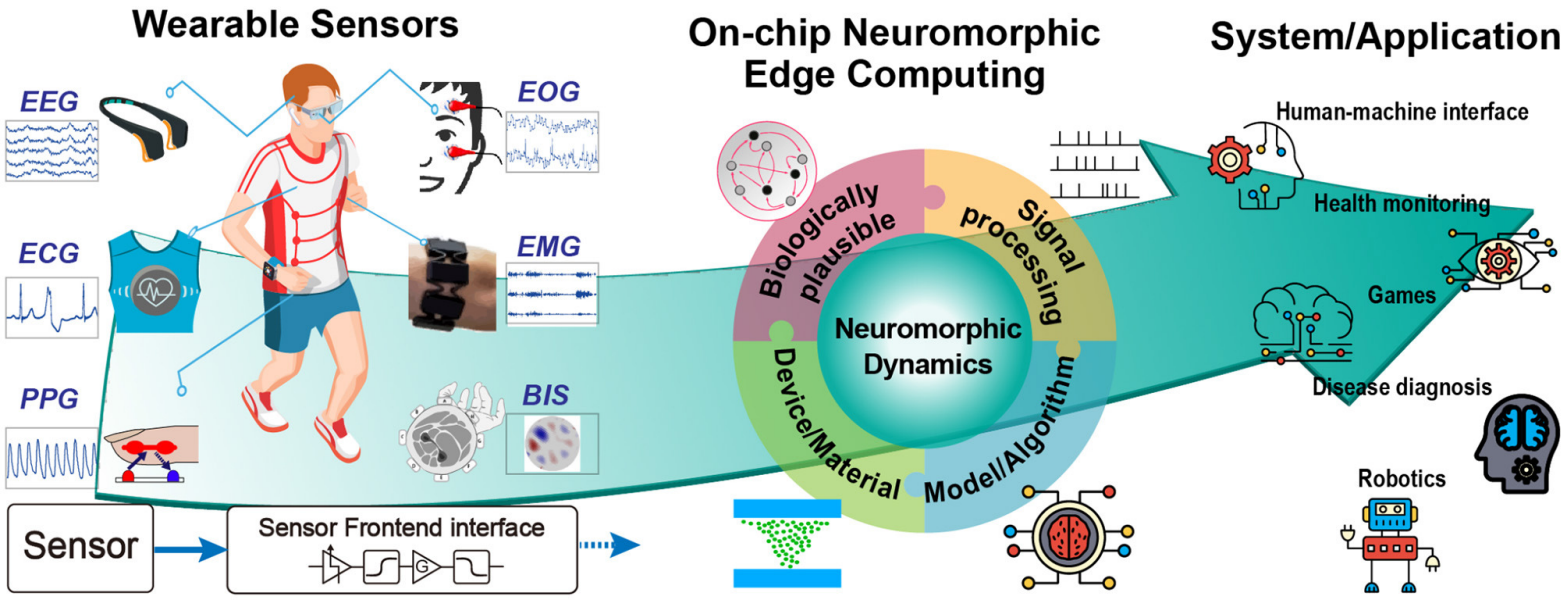
A graphical overview of adaptive edge computing in wearable biomedical devices. The figure shows the pathway from wearable sensors to their application through intelligent learning. EMG and BIS figures adapted from Benalcázar et al. (2017) and Zhang and Harrison (2015).

This review discusses the challenges to undertake for designing extreme edge computing wearable devices for healthcare and biomedical applications in four different categories: (i) the state-of-the-art wearable sensors and main restrictions toward low-power and high performance learning capabilities; (ii) different algorithms for modeling biologically plausible continual learning; (iii) CMOS-based neuromorphic processors and signal processing techniques enabling low-power local edge computing strategies; (iv) emerging memristive devices for more efficient and scalable embedded intelligent systems. We focus on neuromorphic systems as key enabler of extreme edge computing paradigms since they offer a very convenient trade-off between computational capability and power consumption. As graphically summarized in Figure 1, we argue that a holistic approach which combines and exploits all the strengths of these four categories in a co-designed system is the key factor enabling future generations of smart sensing systems.

## 2. Wearable Sensors

Sensors act as the information collector of a machine or a system that can respond to its physical ambient environment. They are able to translate a specific type of information from a physical environment, such as the human body, to an electrical signal (Gao et al., 2016). Wearable devices enable mass ambient data collection from humans and surrounding environment and require miniaturized, flexible, and highly sensitive sensors to capture clear information from the body. However, from processing aspect and to make a signal meaningful toward personalized devices, further development is still needed.

Since the sensing signal is relatively weak and noisy, a readout circuit (normally composed by an amplifier, a conditioning circuit and an analog signal processing unit) is necessary to make the signal readable for a system (Kanoun and Tränkler, 2004; Gao et al., 2016). The subsequent high-level system processes the data and sends commands to actuators for a closed-loop control or interaction (Witkowski et al., 2014; Lopez et al., 2018; Nweke et al., 2018). For various applications ranging from human-machine interfaces (Lopez et al., 2018) to health monitoring (Pantelopoulos and Bourbakis, 2010; Herry et al., 2017), different combinations of sensor and systems have been developed over the past decade (Li et al., 2018c; Liang et al., 2019). The use of machine learning empowers sensors to build novel smart applications. The examples will be provided in this section.

### 2.1. Wearable Sensors With Machine Learning

Recently, the field of artificial intelligence further boosts the possibility of smart wearable sensory systems. The emerging intelligent applications and high-performance systems require more complexity and demand sensory units to accurately describe the physical object. The decision-making unit or algorithm can therefore output a more reliable result (Khezri and Jahed, 2007; Wu et al., 2016; He et al., 2017; Liang et al., 2018, 2019). Depending on the signal acquiring position, Figure 1 illustrates four biopotential sensors and two widely used wearable sensors along with their learning systems and applications, which have also been summarized in Table 1. As evident from Table 1, different sensors have very different specifications in terms of bandwidth and signal amplitude, therefore, the front-end interface needs to be designed taking the sensor features into account. The sensors and systems for the biopotential signal will be introduced first, and the other two wearable sensors will be provided separately. The biopotential signal can be extracted from the human body using a sensor with direct electrode contact. The electrochemical activity of the cells in nervous, muscular, and glandular tissue generates ionic currents in the body. An electrode-electrolyte transducer is needed to convert the ionic current to electric current for the front-end circuit. The electrode that is normally made up of metal can be oxidized by the electrolyte, generating metal ions and free electrons. In addition, the anions in the electrolyte can also be oxidized to neutral atoms and free electrons. These free electrons result in current flow through the electrode. Thus, the surface potential generated by the electrochemical activities in cells can be sensed by the electrode. However, the bio-signals sensed by the electrode are weak and noisy. Before digitizing the collected signals by Analog to Digital Converter (ADC), an analog front-end is essential to provide a readable signal. The design requirements of the front-end for the biopotential electrodes can be summarized as follows: (i) high common mode rejection ratio; (ii) high signal-to-noise-ratio; (iii) low-power consumption; (iv) signal filtering, and (v) configurable gain (Yazicioglu et al., 2008).

**Table 1:** Wearable biomedical signals and sensors.

**Signal**	**Sensor**	**Position**	**Signal band (Hz)**	**Amplitude (mV)**	**Information**	**Application**
ECG	Electrode	Chest	0.5–200	0.05–3	Heart contraction and relaxation	Heartrate monitoring, cardiovascular disease diagnosis
EMG	Electrode	Forearm surface/implant	20–1,000	0.01–10	Muscle activity	Human-machine interaction
EEG	Electrode	Head surface/implant	0.1–100	0.001–0.1	Brain activity	Brain-computer interface, brain disorder monitoring
EOG	Electrode	Around eye	0.1–10	0.001–0.1	Gaze	Human-machine interaction
BIS	Drive electrodes and measurement electrodes	Body	>0.1	–	Body tissue impedance	Cancer detection, health evaluation, human-machine interaction
PPG	Light emitter and receiver	Body	0.1–10	–	Pulse	Heartrate monitoring, biometric identification

#### 2.1.1. Electrocardiography (ECG)

ECG sensor measures the electrical activity generated by the electrochemistry around cardiac tissue. Containing morphological or statistical features, ECG provides comprehensive information for analyzing and diagnosing cardiovascular diseases (Luz et al., 2016; Liang et al., 2020). In previous studies, automatic ECG classification has been achieved using machine learning techniques, such as Deep Neural Network (DNN) (Kiranyaz et al., 2016; Rahhal et al., 2016), Support Vector Machine (SVM) (Zhang et al., 2014; Raj et al., 2016), and Recurrent Neural Network (RNN) (Alfaras et al., 2019; Ortín et al., 2019). According to the Association for the Advancement of Medical Instrumentation, there are five classes of ECG type of interest: normal, ventricular, supraventricular, fusion of normal and ventricular, and unknown beats. These methodologies can be evaluated by available ECG database and yield over 90% accuracy and sensitivity for the five classes, which is essential for future cardiovascular health monitoring. In wearable application, Hossain and Muhammad (2016) and Yang et al. (2016) present systems that measure ECG and send it to the cloud for classification and health monitoring. Furthermore, ECG sensor has been embedded in some of the commercially available devices, such as Apple watch (Apple Inc.), which also enables self-diagnosis for simple cardiovascular disease like atrial fibrillation (Isakadze and Martin, 2020).

#### 2.1.2. Electroencephalography (EEG)

Our brain neurons communicate with each other through electrical impulses. An EEG electrode can help to detect potential information associated with this activity through investigating EEG (Lin et al., 2014; Jebelli et al., 2018) on the surface of the skull. In comparison with other biopotential signals, surface EEG is relatively weak (normally in the range of microvolt-level) and noisy (Gargiulo et al., 2010; Thakor, 2015). Therefore, it requires high input impedance readout circuit and intensive signal pre-processing for clean EEG data (Yazicioglu et al., 2008; Jebelli et al., 2018). While wet-electrode (Ag/AgCl) is more precise and more suitable for clinical purpose, passive dry-electrode is more suitable for daily health monitoring and brain-machine interface (Gargiulo et al., 2010; Li et al., 2015). Besides, the applications also include mental disorder (Shen et al., 2008), driving safety (Lin et al., 2014; Li et al., 2015), and emotion evaluation (Wang et al., 2014b). A commercial biopotential data acquisition system, Biosemi Active Two, provides up to 256 channels for EEG analysis (BioSemi, 2020). For a specific application, we can reduce the number of electrodes to only detect the relevant areas, such as 19 channels for depression diagnosis (Hosseinifard et al., 2013), four channels for evaluating driver vigilance (Lin et al., 2014) and 64 channels for emotional state classification (Wang et al., 2014b). Although EEG is on-body biopotential, most of the existing EEG researches employed offline learning and analysis because of the system complexity and the high number of channels. In wearable real-time applications, a smaller number of channels are usually selected and the data are wirelessly sent to cloud for further processing (Lin et al., 2014; Li et al., 2015; Xu et al., 2017; Hwang et al., 2018).

#### 2.1.3. Electrooculography (EOG)

The eye movement, which results in potential variations around eyes as EOG, is a combined effect of environmental and psychological changes. It returns relatively weak voltage (0.01–0.1 mV) and low frequency (0.1–10 Hz) (Thakor, 2015). Differently from other eye tracking techniques using a video camera and infrared, EOG provides a lightweight, inexpensive and fully wearable solution to access human's eye movement (Duchowski, 2007). It is the most widely used wearable human-machine interface, especially for assisting quadriplegics (Duchowski, 2007). It has been used to control a wheelchair (Eid et al., 2016), control a prosthesis limb (Duvinage et al., 2011; Witkowski et al., 2014), and evaluate sleeping (Piñero et al., 2004; Zhu et al., 2014; Barua et al., 2019). Additionally, recent studies fuse EEG and EOG to increase the degree of freedom of signal and enhance the system reliability, since they have similar implicit information, such as sleepiness (Martin et al., 1972; Barua et al., 2019) and mental health (Stevens et al., 1979). EOG can also act as a supplement to provide additional functions or commands to an EEG system (Punsawad et al., 2010; Wang et al., 2014a; Witkowski et al., 2014).

#### 2.1.4. Electromyography (EMG)

EMG is an electrodiagnostic method for recording and analyzing the electrical activity generated by skeletal muscles. EMG is generated by skeletal muscle movement, which frequently occurs in arms and legs. It yields higher amplitude (up to 10 mV) and bandwidth (20–1,000 Hz) compared to the other biopotentials (Yazicioglu et al., 2008; Thakor, 2015). Near the active muscle, different oscillation signals can be measured by a dry electrode array, which allows the computer to sense and decode body motion (Rissanen et al., 2008; Wang et al., 2010; Mendez et al., 2017). A prime example is the Myo armband of Thalmic Labs, which is a commercial multi-sensor device that consists of EMG sensors, gyroscope, accelerometer and magnetometer (Rawat et al., 2016). The sensory data is sent to phone or PC via Bluetooth, where various body movements can be classified by feature extraction and machine learning techniques. Moreover, the application of EMG is frequently linked to target control like a wheelchair (Inhyuk et al., 2005) and prosthetic hand (Cipriani et al., 2008; Artemiadis and Kyriakopoulos, 2011) for assisting disabled people. In addition, its application also includes sign language recognition (Mendez et al., 2017), diagnosis of neuromuscular disorders (Rissanen et al., 2008; Subasi, 2013), analysis of walking strides (Wang et al., 2010), and virtual reality (Rincon et al., 2016). Machine learning enables the system to overcome the variation of EMG signals from different users (Rissanen et al., 2008; Mendez et al., 2017).

#### 2.1.5. Photoplethysmography (PPG)

PPG is an non-invasive and low-cost optical measurement method that is often used for blood pressure and heart rate monitoring in wearable devices. The optical properties in skin and tissue are periodically changing due to the blood flow driven by the heartbeat. By using a light emitter toward the skin surface, the photosensor can detect the variations in light absorption, normally from wrist or finger. This signal variation is called PPG, which is highly relevant to the rhythm of the cardiovascular system (Biswas et al., 2019b). Compared with ECG, PPG is easily accessible and low cost, which makes it an ideal intermedia of wearable heart rate measurement. Wrist-PPG has already been deployed in various commercial smartwatches or wristbands, such as Apple Watch, Fitbit Charge, and TomTom Touch, for heart-rate monitoring (Hough et al., 2017). The main disadvantage against ECG is that the PPG is relatively less informative and not unique for different persons and body positions. Thus, further analysis of PPG requires machine learning or other statistics tools for calibrating the signal to different scenarios. For example, it can be used in biometric identification after deep learning (Reşit Kavsaoğlu et al., 2014; Biswas et al., 2019a). It is worth mentioning that PPG can be also a strong supplementary indicator in the application of ECG.

#### 2.1.6. Bioimpedance spectroscopy (BIS)

BIS is another low-cost and powerful sensing technique that provides informative body parameters. The principle is that cell membrane behaves like a frequency-dependent capacitor and impedance. The emitter electrodes generate multifrequency excitation signal (0.1–100 MHz) on the skin while the receiver electrodes collect these currents for demodulating the impedance spectral data of the tissue in between (Matthie, 2008; Caytak et al., 2019). Compared to homogeneous materials, body tissue presents more complicated impedance spectra due to the cell membranes and macromolecules. Therefore, the tissue conditions, such as muscle concentration, structural, and chemical composition, can be analysed through BIS. The BIS can measure body composition, such as fat and water (Matthie, 2008). Based on the different setup in terms of position and frequency, it can also be helpful in the early detection of diseases, such as lymphedema, organ ischemia, and cancer (Sun et al., 2010). Furthermore, multiple pair-wise electrodes can form electrical impedance tomography that describes impedance distribution. By embedding these electrodes in a wristband, the tomography can estimate hand gestures after training, which is another novel solution of inexpensive human-machine interface (Zhang et al., 2016).

### 2.2. Multisensory Fusion in Wearable Devices

Every sensor has its own limitation. In some demanding cases, a single sensor itself cannot satisfy the system requirement, such as accuracy or robustness (Khaleghi et al., 2013; Alsheikh et al., 2014; Gravina et al., 2017; Liang et al., 2019). The solution involves increasing the number and type of sensors to form a multisensory system or sensor network for one measured target (Khaleghi et al., 2013; Alsheikh et al., 2014; Gravina et al., 2017). Multiple types of sensor synergistically working in a system provide more dimensions of input to fully map an object onto the data stream. Different sensors return different data with respect to sampling rate, number of inputs and the information behind the data. Machine learning models, such as ANN and SVM, can be designed to combine multiple sources of data. Depending on the application, sensor types and data structure, several approaches have been proposed for multisensory fusion. Generally, in such a system, machine learning is frequently used and plays a vital role in merging different sources of sensory data based on its multidimensional data processing mechanism. The machine learning algorithms enable sensory fusion to occur at the signal, feature or decision level (Khaleghi et al., 2013; Gravina et al., 2017). When dealing with SNN, the multi-sensory features or raw-data need to be encoded and fused in spike sequences in order to fit the input modality of the spike-based neural network. Furthermore, encoding the information in spikes can also further attenuate the risk of catastrophic forgetting issue in conventional neural networks (Azghadi et al., 2020). For decision level fusion, a voting mechanism is typically needed to output the final result after receiving the decisions from different sources of sensors which may be processed by different networks with different algorithms (Li et al., 2017). The results showed that a multisensory system is advantageous in improving system performance. For example, the fusion of ECG and PPG patterns can be an informative physiological parameter for robust medical assessment (Rundo et al., 2018). Counting the peak intervals between PPG and ECG can estimate the arterial blood pressure (He et al., 2014). Interestingly, a recent study shows that the QRS complex of ECG can be reconstructed from PPG by a novel transformed attentional neural network after training (Chiu et al., 2020). This could be beneficial for the accessibility of wearable ECG.

### 2.3. Challenges Toward Smart Wearable Sensors With Edge Computing

The novel applications using multiple sensors and high learning ability usually require more energy in the wearable computing unit (Pantelopoulos and Bourbakis, 2010). Nevertheless, the power supply in the wearable domain is a difficulty with existing battery technologies. This weakness limits the further development of smart wearable devices (Pantelopoulos and Bourbakis, 2010). The existing solution is to wirelessly transfer the raw data onto a cloud where the computationally intensive algorithm is implemented (Patel et al., 2016). However, this solution is not ideal considering (i) the complexity of using a wireless module, (ii) the non-negligible power consumption, (iii) the amount of data, (iv) the space limitation due to the range of wireless transmission, (v) privacy issues due to the broadcast of signals, and (vi) non-negligible time latency due to communication channel. These technological drawbacks strongly limit the application of wearable sensors.

Implementations of ANN in von Neumann architectures, which have been frequently used in sensors, result therefore in a non-optimized distribution of the energy consumption. Conversely, it has been reported that signal processing activity in the brain is several orders of magnitudes more power-efficient and one order in processing rate better than digital systems (Mead, 2020). Compared to conventional approaches based on a binary digital system, brain-inspired neuromorphic hardware has yet to be advanced in the contexts of data storage and removal as well as their transmission between different units. In this perspective, a neuromorphic chip with a built-in intelligent algorithm can act as a front-end processor next to the sensor. The conventional ADCs could be replaced by a delta encoder or feature extractor converting the sensor analog output to spike-based signal for the hardware (see Section 4). In the end, the output becomes the result of recognition or prediction instead of an intensive data stream. In this way, the computation occurs at the local edge under low power and brain-like architecture. In summary, the research on on-chip neuromorphic edge computing is a multidisciplinary topic involving biologically plausible algorithms, device/material engineering, system modeling/co-design, and signal processing (Figure 1). The following sections will provide more comprehensive discussion toward these subjects.

## 3. Algorithms for Biologically Plausible Continual Learning

In this section we will highlight some recently introduced methods to port the power of modern machine learning to neuromorphic edge devices. In the last couple of years, machine learning has made big steps forward reaching close-to human performance on a wide range of tasks. Many of the most successful machine learning methods are based on artificial neural networks (ANN), which are inspired by the organization of information processing in the brain. However, somewhat contradictory—mapping modern ANN learning methods to brain-inspired hardware poses considerable challenges to the algorithm and hardware design. The main reason for this is, that the development of machine learning algorithms has been strongly influenced by the development of powerful mainframe computers that perform learning offline in big server farms only eventually sending back results to the user. While this development has paved the ground for today's success of ANNs, it has also lead the field away from following the principles used in biology for efficient learning.

Neuromorphic realizations of on-chip learning have therefore often focused on biologically inspired learning rules, such as Spike-Timing Dependent Plasticity (STDP). In this model, synaptic weight changes only take place if pre-synaptic spikes arrive at the synapse, which makes them very well-suited for event-based algorithms (Diehl and Cook, 2014; Chen et al., 2018; Li et al., 2018b; Lin et al., 2018). In this section we focus on algorithmic advances that combine the efficiency of bio-inspired plasticity rules with modern machine learning approaches. In the following section 3.1 we will review recent approaches to combine the strengths of modern machine learning and brain-inspired algorithms, that are of particular interest for edge computing applications. In section 3.2 we will focus on the problem to cope with extreme memory constraints by exploiting sparsity. In section 3.3 we will highlight additional open challenges and future work.

### 3.1. Brain-Inspired Learning Algorithms for Neuromorphic Hardware

Today, the dominating method for training artificial neural networks is the error backpropagation (Backprop) algorithm (Rumelhart et al., 1986), which provides an efficient and scalable solution to adapting the network parameters to a set of training data. Backprop is an iterative, gradient-based, supervised learning algorithm that operates in three phases. First, a given input activation is propagated through the network to generate the output based on the current set of parameters. Then, the mismatch between the generated outputs and target values is computed using a loss function, and propagated backwards through the network architecture to compute suitable weight changes. Finally, the network parameters are updated to reduce the loss. We will not go into the details behind Backprop here, but see Schmidhuber (2015) for an excellent review and historical survey of the development of the algorithm. The problem of porting Backprop to neuromorphic hardware stems from a well-known shortcoming of the algorithm known as *locking* (Czarnecki et al., 2017). The weights of a network can only be updated after a full forward propagation of the data through the network, followed by loss evaluation. A learning cycle ends after waiting for the back-propagation of error gradients, which makes an efficient implementation of Backprop on online distributed architectures challenging. Also, Backprop is not well-suited for spiking neural networks which have non-differentiable output functions. These problems have been recently addressed in brain-inspired variants of the Backprop algorithm.

#### 3.1.1. Brain-Inspired Alternatives to Error Backpropagation

In recent years a number of methods have been proposed to approximate the gradient computation performed by Backprop in order to prevent locking (see Richards et al., 2019 for a recent review). Lillicrap et al. (2016) and Samadi et al. (2017) proposed to replace the non-local error back-propagating term of the Backprop algorithm by sending the loss through a fixed feedback network with random weights that are excluded from training. In this approach, named *random feedback alignment* the back-propagating error signal acts as a local feedback to each synapse, similar to a reward signal in reinforcement learning. The fixed random feedback network de-correlates the error signals providing individual feedback to each synapse. Lillicrap et al. could show that this simple approach already provides a viable approximation to the exact Backprop algorithm and performs well for practical machine learning problems of moderate size. In Neftci et al. (2017) an event-based version of random feedback alignment, that is well-suitable for neuromorphic hardware, was introduced. This approach was further generalized in Payvand et al. (2020a) to include a larger class of algorithms that use error feedback signals.

An efficient model for learning complex sequences in spiking neural networks, named *Superspike*, was introduced in Zenke and Ganguli (2018). The model also uses a learning rule that is modulated by error feedback signals and locally minimizes the mismatch between the network output and a target spike train. To overcome the problem of non-differentiable output, Superspike uses a surrogate gradient approach that replaces the infinitely steep spike events with a finite auxiliary function at the time points of network spike events (Bengio et al., 2013). As in random feedback alignment, learning signals are communicated to the synapses via a feedback network with fixed weights. Using this approach Zenke and others could demonstrate efficient learning of complex sequences in spiking networks.

Another approach to approximate Backprop in spiking neural networks uses an anatomical detail of Cortical neurons. Sacramento et al. (2017) introduced a biologically inspired two-compartment neuron model that approximates the error backpropagation algorithm by minimizing a local dendritic prediction error. Göltz et al. (2019) port learning by Backprop to neuromorphic hardware by incorporating dynamics with finite time constants and by optimizing the backward pass with respect to substrate variability. They demonstrate the algorithm on the BrainScaleS analog neuromorphic architecture.

#### 3.1.2. Brain-Inspired Alternatives to Backpropagation Through Time

**Figure 2:**
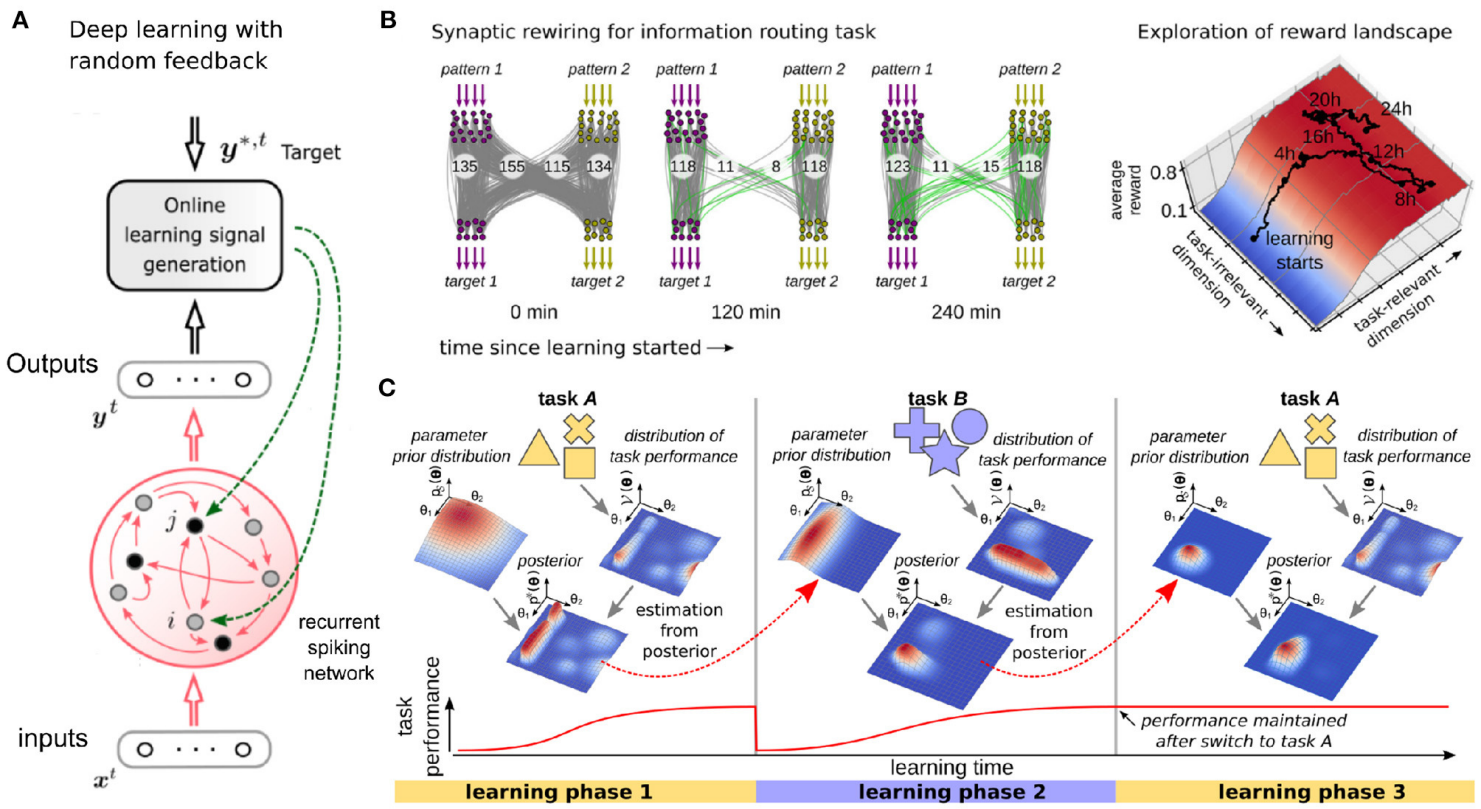
Biologically inspired algorithms of learning in spiking neural networks. **(A)** The e-prop algorithm (Bellec et al., 2019) approximates back-propagation through time using random feedback to propagate error signals to synapses of a recurrent SNN (adapted from Bellec et al., 2020). **(B)** Synaptic sampling (Kappel et al., 2015) exploits the variability of learning rules and redundancy in the task solution space to learn sparse and robust network configurations (adapted from Kappel et al., 2018). **(C)** Overcoming forgetting by selectively slowing down weight changes (Kirkpatrick et al., 2017). After learning a first task A, parameter distributions are absorbed into a prior distribution that confines the motility of synaptic weights in subsequent tasks (task B).

Recurrent neural network (RNN) architectures often show superior learning results for tasks that involve a temporal dimension, which is often the case for edge computing applications. Porting learning algorithms for RNNs is therefore of utmost importance for efficient machine learning on the edge. Backpropagation through time (BPTT)—the standard RNN learning method used in most GPU implementations—unfolds the network in time and keeps this extended structure in memory to propagate information forward and backward which poses a severe challenge to the power and area constraints of edge computing. Recent theoretical results (Bellec et al., 2018, 2019) show that the power of BPTT can be brought to biologically inspired spiking neural networks (SNN) while at the same time the unfolding can be prevented in an approximation that operates only forward in time, enabling *online, always-on* learning. This algorithm operates at every synapse in parallel and incrementally updates the synaptic weights. As for random feedback alignment and Superspike discussed above, the weight update depends only on three factors, where the first two are determined by the states of the two related input/output neurons, and the third is given by synapse-specific feedback conveying the mismatch between the target and the actual output (see Figure 2A for an illustration). The temporal gap between these factors is mitigated by an *eligibility trace* describing a transient dynamic. Eligibility traces, have been theoretically predicted for a long time (Williams, 1992; Izhikevich, 2007), and have also recently been observed experimentally in the brain (Yagishita et al., 2014; Brzosko et al., 2015; He et al., 2015; Bittner et al., 2017).

### 3.2. Efficient Learning Under Stringent Memory Constraints

The amount of available resources in neuromorphic systems is kept low to increase energy efficiency. Memory elements are especially impactful on the energy budget. Therefore, algorithms are needed that make efficient use of the available memory resources. The largest amount of memory in a network is usually consumed by the synaptic weights. Since in practice, the weights of many connections in a network converge to values close to zero, several methods have been proposed to reduce the memory footprint of machine learning algorithms by exploiting sparsity in the network connectivity. Also in many applications the bit precision per synapse can be reduced without significant performance loss which further reduces the memory footprint. We will discuss here three types of algorithms that work under stringent memory constraints: (i) those that are based on *pruning connections after learning*, (ii) *online* learning with *sparse* networks and (iii) *quantization-aware training* that implements learning algorithms in networks with reduced bit precision per weight.

#### 3.2.1. Pruning

Many approaches to exploit sparsity in learning algorithms focus on pruning the network after training (see Gale et al., 2019 for a recent review). Simple methods rely on pruning by magnitude, simply by eliminating the weakest (closest to zero) weights in the network (Ström, 1997; Collins and Kohli, 2014; Han et al., 2015). Some methods based on this idea have reported impressive sparsity rates of over 95% for standard machine learning benchmarks with negligible performance loss (Guo et al., 2016; Zhu and Gupta, 2017). Other methods are based on theoretical motivations and classical sparsification and regularization techniques (Louizos et al., 2017; Molchanov et al., 2017; Ullrich et al., 2017). These models reach high compression rates. Dai et al. (2019) proposed a method to iteratively grow and prune a network in order to generate a compact yet precise solution. They provide a detailed comparison with state of the art dense networks and other pruning methods and reaching sparsity above 99% for the LeNet-5 benchmark.

#### 3.2.2. Online Learning in Sparse Networks

A number of authors also introduced methods that work directly with sparse networks during training, which is often the more interesting case for neuromorphic applications with online training. Bellec et al. (2017) introduced an algorithm for online stochastic rewiring in deep neural networks that works with a fixed number of synaptic connections throughout learning. The algorithm showed close-to state of the art performance at up to 98% sparsity. Sparse evolutionary training (SET) (Mocanu et al., 2018) introduced a heuristic approach that prunes the smallest weights and regrows new weights in random locations. Dynamic Sparse Reparameterization (Mostafa and Wang, 2019) introduces a prune-redistribute-regrowth cycle. They demonstrated compelling performance levels also for very deep neural network architectures. Lee et al. (2018) introduced a single shot pruning algorithm that yields sparse networks based on a saliency criterion prior to the actual training. Dettmers and Zettlemoyer (2019) introduced a refined method for online pruning and redistribution that surpasses the previous methods in terms of sparsity and learning performance.

#### 3.2.3. Quantization-Aware Training

Quantization-aware training is today a common method applied in commercial and practical settings to port machine learning to hardware with reduced bit precision per synapse. Several approaches have been proposed. *Stochastic rounding* translates the weight update into a probability and flips the weights to the closest quantized value. This method has been applied to online and offline learning with very low bit resolutions of down to 2 bits per synapse (Müller and Indiveri, 2015; Müller et al., 2017). (Hubara et al., 2016) introduced a *binary deep neural network* architecture that uses only two weight values (+1 and −1), achieving compelling learning performance. The weight quantization was implemented with smooth functions so that widely available implementations of error Backpropagation could be used. Wang et al. (2018a) and Sun et al. (2019) demonstrated deep learning in large state-of-the-art networks with 8-bit precision floating point weights. Finally in recent work (Choi et al., 2020) regularization, quantization and pruning was combined to train compressed deep learning models and a detailed performance analysis was provided.

### 3.3. Open Challenges and Future Work

As outlined above, edge computing poses quite specific challenges to learning algorithms that are substantially different from requirements of classical applications. Some of the algorithms outlined above have already been successfully ported to neuromorphic hardware. For example, the e-prop algorithm of Bellec et al. (2018) has been implemented on the SpiNNaker 2 chip yielding an additional energy reduction by two orders of magnitude compared to a X86 implementation (Liu et al., 2018). See the next section 4 for more details on available neuromorphic hardware and their applications.

In the remainder of this section we will highlight open challenges that remain to be solved for efficient learning in edge computing applications. In addition to the stringent memory and power constraints learning at the edge also has to function in an online scenario where data arrive in a continuous stream. Some dedicated hardware resources, e.g., like memristive devices discussed in section 5, may also show high levels in intrinsic variability, so the learning algorithm should be robust against these noise sources. In this section we discuss recent advances in this line of research and provide food for thought on how these specific challenges can be approached in future work.

#### 3.3.1. Robust Learning Algorithms for Neuromorphic Devices Exploiting Device Noise

Here we review recent advances in using inspiration from biology to make learning algorithms robust against device variability. Several authors have suggested that device noise and variability should not be seen as a nuisance, but rather can serve as a computational resource for network simulation and learning algorithms (see Maass, 2014 for a thorough discussion). Pecevski and Maass (2016) have shown that variability in neuronal outputs can be exploited to learn complex statistical dependencies between sensory stimuli. The stochastic behavior of the neurons is used in this model to compute probabilistic inference, while biologically motivated learning rules, that only require local information at the synapses can be used to update the synaptic weights. A theoretical foundation of the model shows that the spiking network performs a Markov chain Monte Carlo sampling process, that allows the network to “reason” about statistical problems.

This idea is taken one step further in Neftci et al. (2015) by showing that also the variability of synaptic transmission can be used for stochastic computing. The intrinsic noise of synaptic release is used to drive a sampling process that can be implemented in an event-based fashion. In Kappel et al. (2015) it was shown that the variability of learning rules and weight parameters gives rise to a biologically plausible model of online learning. The intrinsic noise of synaptic weight changes drives a sampling process that can be used to exploit redundancies in the task solution space (see Figure 2B for an illustration). This model was applied to unsupervised learning in spiking neural networks, and to closed-loop reinforcement learning problems (Kappel et al., 2018; Kaiser et al., 2019). In Yan et al. (2019) this model was also ported to the SpiNNaker 2 neuromorphic many-core system.

#### 3.3.2. Biologically Motivated Mechanisms to Combat Forgetting in Always-on Learning Scenarios

Neuromorphic systems often operate in an environment where they are permanently on and learning a continuous stream of data. This mode of operation is quite different from most other machine learning applications that work with hand-labeled batches of training data. Always-on learning inevitably leads to forgetting previously learned sensory experiences as a necessary consequence of applying weight updates over time (Fusi et al., 2005; Benna and Fusi, 2016). Inspiration to solve the associated stability-plasticity problem by protecting relevant information comes from biology. The mammalian brain seems to combat forgetting relevant memories by actively protecting previously acquired knowledge in neocortical circuits (Pan and Yang, 2009; Yang et al., 2009, 2014; Cichon and Gan, 2015; Hayashi-Takagi et al., 2015). When a new skill is acquired, a subset of synapses is strengthened, stabilized and persists despite the subsequent learning of other tasks (Yang et al., 2009).

A theoretical treatment of the forgetting problem was conducted in the *cascade model* of Stefano Fusi and others (Fusi et al., 2005; Benna and Fusi, 2016). They could show that learning an increasing number of patterns in a single neural network leads unavoidably to a state which they called catastrophic forgetting. Trying to train more patterns into the network will interfere with all previously learned ones, effectively wiping out the information stored in the network. The proposed cascade model to overcome this problem uses multiple parameters per synapse that are linked through a cascade of local interactions. This cascade of parameters selectively slows down weight changes, thus stabilizes synapses when required and effectively combats effects of catastrophic forgetting. A related model, that uses multiple parameters per synapse to combat forgetting was used in Kirkpatrick et al. (2017) (see also Huszár, 2018 for a recently introduced variation of the model). They used a Bayesian approach that infers a prior distribution over parameter values at each synapse. Synapses that stabilize during learning (converge to a fixed solution) will be considered relevant in subsequent learning and Bayesian priors help to maintain their values (see Figure 2C for an illustration).

Another promising biologically inspired method that has recently gained attention in machine learning, and which may enable a system to benefit from large amounts of unlabeled data, is *self-supervised learning*. This technique augments the learning problem with pretext tasks which can be formulated using only unlabeled data, but do require higher-level semantic understanding in order to be solved (Hendrycks et al., 2019; Zhai et al., 2019). These pretext tasks typically involve a simple manipulation of the input, such as image rotation, for which a target objective can be computed without supervision (Kolesnikov et al., 2019). A promising recent approach combines self-supervised learning and semi-supervised learning where sparse labeled data is used to enhance the model performance (Zhai et al., 2019). This method that incorporates sparse feedback from a supervisor might be of particular interest for edge devices.

#### 3.3.3. Biologically Motivated Mechanisms to Enhancing Transfer and Sensor Fusion

Distributed computing architectures at the edge need to make decisions by integrate information from different sensors and sensor modalities and should be able to best make use of the sensory information across a wide range of tasks. It is clearly not very efficient to learn from scratch when confronted with a new task. Therefore, to boost the performance of edge computing, we consider here two aspects of transferring information to new situations: transfer of knowledge between sensors (*sensor fusion*), which has been treated in section 2.2, and transfer of knowledge between multiple different tasks (*transfer learning*).

*Transfer learning* denotes the improvement of learning in a new task through the use of knowledge from a related task that has already been learned previously (Caruana, 1997; Torrey and Shavlik, 2010). This contrasts most other of today's machine learning applications that focus on one very specific task. In transfer learning, when a new task is learned, knowledge from previous skills can be reused without interfering with them. For example, the ability to perform a tennis swing can be transferred to playing ping pong, while maintaining the ability to do both sports. The literature on transfer learning is extensive and many different strategies have been developed depending on the relationship between the different task domains (see Lu et al., 2015 and Weiss et al., 2016 for systematic reviews). In machine learning a number of approaches have been applied to a wide range of problems, including classification of images (Kulis et al., 2011; Zhu et al., 2011; Duan et al., 2012; Long et al., 2017), text (Prettenhofer and Stein, 2010; Wang and Mahadevan, 2011; Zhou et al., 2014a,b), or human activity (Harel and Mannor, 2010).

A very general approach to learn across multiple domains is followed in the *learning to learn* framework of Schmidhuber (1992, 1993). Their model features networks that are able to modify their own weights through the network activity. These network are therefore able to tinker with their own processing properties. This approach has been taken to its most extreme form where a network leans to implement an optimization algorithm by itself (Andrychowicz et al., 2016). This model consists of an outer-loop learning network (*the optimizer*) that controls the parameters of an inner-loop network (*the optimizee*). The training algorithm of the inner-loop network works on single tasks that are presented sequentially, whereas the outer-loop learner operates across tasks and can acquire strategies to transfer knowledge. This learning-to-learn framework was recently applied to SNNs to obtain properties of LSTM networks and use them to solve complex sequence learning tasks (Bellec et al., 2018). In Bohnstingl et al. (2019), the learning-to-learn framework was also applied to a neuromorphic hardware platform.

## 4. Signal Processing for Wearable Devices on Neuromorphic Chip

Neuromorphic engineering is a branch of electrical engineering dedicated to the design of analog/digital data processors that aims to emulate biological neurons and synapses. The key technological advantage of neuromorphic chips lies in (i) their power efficiency as a result of reducing data movement through co-location of memory and processor and sparsifying the temporal information through events (spikes); (ii) their low latency since they enable the real-time processing of signals through temporal dynamics and (iii) their adaptive properties which enable adjusting their parameters to the environment they are being employed.

This increasing interest in neuromorphic engineering shows that hardware SNNs are considered a key future technology with high potential in key application, such as edge computing, and wearable devices.

Neuromorphic technologies have sparked interest from universities (Furber et al., 2014; Qiao et al., 2015; Moradi et al., 2017; Neckar et al., 2018; Schemmel et al., 2020) and companies, such as IBM (Merolla et al., 2014) and Intel (Davies et al., 2018). There are two main approaches of fully-digital and analog/digital mixed-signal that have been taken to design event-driven neuromorphic chips. The similarities between the two types are the employment of events and sending packets for communicating information between different computational cores. The employed communication scheme is Address-Event Representation (AER), where the communicating neurons place their address on a shared communication bus whenever they spike. The difference between the two approach is the way the computation is done. In the digital approach, the Vector Matrix Multiplication (VMM) and the dynamics are calculated using bit-precise and time-stepped approach, whereas in the mixed-signal approach the physics of the computational substrate is used.

In this section, we will provide an overview of the neuromorphic platforms, that to the best of our knowledge were deployed for biomedical signal processing, showing promising results to be exploited in wearable devices.

### 4.1. Neuromorphic Processors

#### 4.1.1. TrueNorth

TrueNorth (Merolla et al., 2014) is IBM's neuromorphic chip that uses a digital approach for both processing and communication. One million neurons arranged in a tiled array of 4,096 neurosynaptic cores enable *massive parallel processing*. Each core contains 13 kB of *local SRAM memory* to keep neurons and synapse's states along with the axonal delays and information on the fan-out destination. There are 256 Leaky-Integrator and Fire (LIF) neurons implemented by time-multiplexing and 256 million synapses are designed in the form of SRAM memory. Each core can support up to 256 fan-in and fan-out, and this connectivity can be configured such that a neuron in any core can communicate its spikes to any other neuron in any other core.

Thanks to the *event-driven nature*, the co-location of memory and processing units in each core, and the use of low-leakage silicon CMOS technology, TrueNorth can perform 46 billion synaptic operations per second (SOPS) per watt for real-time operation, with 26 pJ per synaptic event. Its power density of 20 mW/cm^2^ is about three orders of magnitude smaller than that of typical CPUs.

#### 4.1.2. SpiNNaker

The SpiNNaker machine (Furber et al., 2014), designed by the University of Manchester, is a custom-designed ASIC based on *massively parallel architecture* that has been designed to efficiently simulate large spiking neural networks. It consists of ARM968 processing cores arranged in a 2D array where the precise details of the neurons and their dynamics can be programmed. Although the processing cores are synchronous microprocessors, the *event-based* aspect of SpiNNaker is apparent in its message-handling paradigm. A message (event) gets delivered to a core generating a request for being processed. The communications infrastructure between these nodes is specially optimized to carry very large numbers of very small packets, optimal for spiking neurons.

A second generation of SpiNNaker was designed by Technical University of Dresden (Mayr et al., 2019). Spinnaker2 continues the line of dedicated digital neuromorphic chips for brain simulation increasing the simulation capacity by a factor >10 while staying in the same power budget (i.e., 10× better power efficiency). The full-scale SpiNNaker2 consists of 10 Million ARM cores distributed across 70,000 Chips in 10 server racks. This system takes advantage of advanced 22 nm FDSOI technology node with Adaptive Body Biasing enabling reliable and ultra-low power processing. It also features incorporating numerical accelerators for the most common operations.

#### 4.1.3. Loihi

Loihi (Davies et al., 2018) is Intel's neuromorphic chip with many-core processing incorporating on-line learning designed in 14 nm FinFET technology. The chip supports about 130,000 neurons and 130 million synapses distributed in 128 cores. Spikes are transported between the cores in the chip using packetized messages by an asynchronous network on chip. It includes three embedded ×86 processors and provides a very flexible learning engine on which diverse online learning algorithms, such as Spike-Timing Dependent Plasticity (STDP), different three factor and trace-based learning rules can be implemented. The chip also provides hierarchical connectivity, dendritic compartments, synaptic delays as different features that can enrich a spiking neural network. The synaptic weights are stored on local SRAM memory and the bit precision can vary between 1 and 9 bits. All logic in the chip is digital, functionally deterministic, and implemented in an asynchronous bundled data design style.

#### 4.1.4. DYNAP-SE

DYNAP-SE implements a multi-core neuromorphic processor with scalable architecture fabricated using a standard 0.18 μ*m* CMOS technology (Moradi et al., 2017). It is a full-custom asynchronous mixed-signal processor, with a fully asynchronous inter-core and inter-chip hierarchical routing architecture. Each core comprises 256 adaptive exponential integrate-and-fire (AEI&F) neurons for a total of 1k neurons per chip. Each neuron has a Content Addressable Memory (CAM) block, containing 64 addresses representing the pre-synaptic neurons that the neuron is subscribed to. Rich synaptic dynamics are implemented on the chip by using Differential Pair Integrator (DPI) circuits (Bartolozzi and Indiveri, 2007). These circuits produce EPSCs and IPSCs (Excitatory/Inhibitory Post-Synaptic Currents), with time constants that can range from a few μ*s* to hundreds of *ms*. The analog circuits are operated in the sub-threshold domain, thus minimizing the dynamic power consumption, and enabling implementations of neural and synaptic behaviors with biologically plausible temporal dynamics. The asynchronous CAMs on the synapses are used to store the tags of the source neuron addresses connected to them, while the SRAM cells are used to program the address of the destination core/chip that the neuron targets.

#### 4.1.5. ODIN/MorphIC

Online-learning DIgital spiking Neuromorphic (ODIN) processor occupies an area of only 0.086 mm^2^ in 28 nm FDSOI CMOS (Frenkel et al., 2019a). It consists of a single neurosynaptic core with 256 neurons and 256^2^ synapses. Each neuron can be configured to phenomenologically reproduce the 20 Izhikevich behaviors of spiking neurons (Izhikevich, 2004). The synapses embed a 3-bit weight and a mapping table bit that allows enabling or disabling Spike-Dependent Synaptic Plasticity (SDSP) locally (Brader et al., 2007), thus allowing for the exploration of both off-chip training and on-chip online learning setups.

MorphIC is a quad-core digital neuromorphic processor with 2k LIF neurons and more than 2M synapses in 65 nm CMOS (Frenkel et al., 2019b). MorphIC was designed for high-density large-scale integration of multi-chip setups. The four 512-neuron crossbar cores are connected with a hierarchical routing infrastructure that enables neuron fan-in and fan-out values of 1k and 2k, respectively. The synapses are binary and can be either programmed with offline-trained weights or trained online with a stochastic version of SDSP.

### 4.2. Biomedical Signal Processing on Neuromorphic Hardware

**Figure 3:**
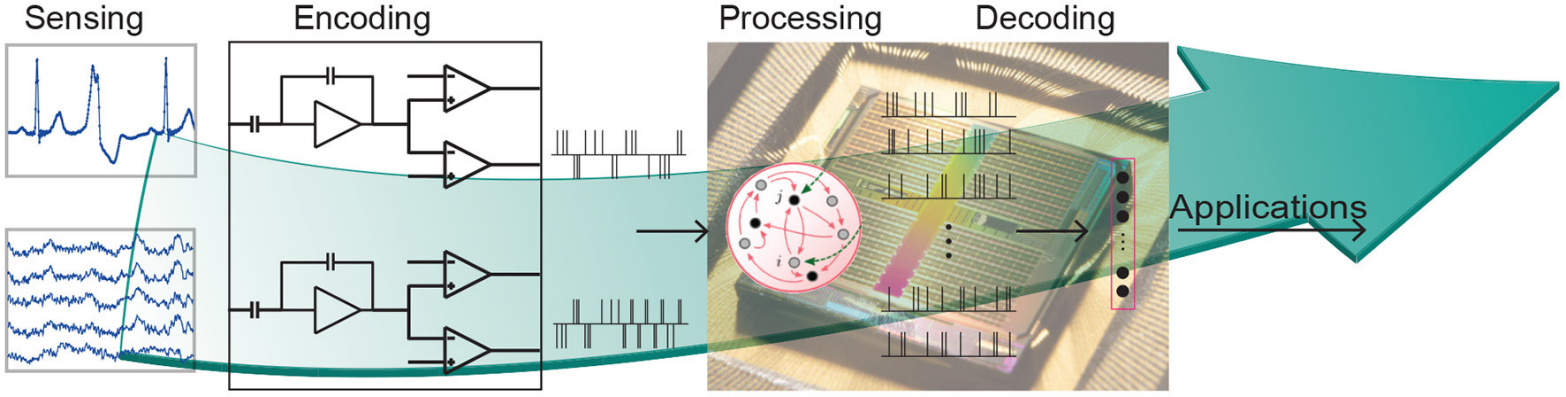
Biomedical signal processing on neuromorphic hardware, from sensors to applications.

Given the low latency and low power properties of these neuromorphic chips, they are promising candidates for on-edge processing of biomedical signals. Figure 3 illustrates the different stages of biomedical processing using a neuromorphic system pipeline. The sensory signals should first be encoded to spikes or events which are fed to a neuromorphic SNN processor. Depending on the application, appropriate SNN architecture is mapped onto the chip and the output (e.g., anomaly detection, or gesture recognition) is read out.

#### 4.2.1. Encoding

In SNNs a single spike by itself does not carry any information. However, the number and the timing of spikes produced by a neuron are important. Just as their biological counterpart, silicon neurons in neuromorphic devices produce spike trains at a rate that is proportional to their input current. At the input side, synapse circuits integrate the spikes they receive to produce analog currents, with temporal dynamics and time constants that can be made equivalent to their biological counterparts. The sum of all the positive (excitatory) and negative (inhibitory) synaptic currents afferent to the neuron is then injected into the neuron.

To provide biomedical signals to the synapses of the SNN input layer, it is necessary to first convert them into spikes. A common way to do this is to use a delta-modulator circuit (Corradi and Indiveri, 2015; Sharifshazileh et al., 2019) functionally equivalent to the one used in the Dynamic Vision Sensor (DVS) (Lichtsteiner et al., 2008). This circuit, in practice, is an ADC that produces two asynchronous digital pulse outputs (UP or DOWN) for every biosignal channel in the input. The UP (DOWN) spikes are generated every time the difference between the current and previous value exceeds a pre-defined threshold. The sign of the difference corresponds to the UP or DOWN channel where the spike is produced. This approach was used to convert EMG signals, used in mixed-signal neuromorphic chips (Donati et al., 2018, 2019) and in digital ones (Behrenbeck et al., 2019; Ceolini et al., 2020), ECG signals (Bauer et al., 2019; Corradi et al., 2019), and EEG and High Frequency Oscillation (HFO) ones (Corradi and Indiveri, 2015; Sharifshazileh et al., 2019).

#### 4.2.2. Processing and Decoding

Table 2 shows the summary of neuromorphic processors described previously where biomedical signal processing applications were used. These works show promising results for always-on embedded biomedical systems.

**Table 2:** Summary of neuromorphic platforms and biomedical applications.

**Neuromorphic chip**	**DYNAP-SE**	**SpiNNaker**	**Loihi**	**TrueNorth**	**ODIN**
CMOS technology	180 nm	ARM968, 130 nm	14 nm FinFET	28 nm	28 nm FDSOI
Implementation	Mixed-signal	Digital	Digital ASIC	Digital ASIC	Digital ASIC
Energy per SOP	17 pJ @ 1.8 V	Peak power 1 W per chip	23.6 pJ @ 0.75 V	26 pJ @ 0.775	12.7 pJ@0.55 V
Size	38.5 mm^2^	102 mm^2^	60 mm^2^	0.093 mm^2^ (core)	0.086 mm^2^
On-chip learning	No	Yes (configurable)	Yes (configurable)	No	Yes (SDSP)
Synaptic bit precision	2	Configurable	1–9	1	3
Applications	EMG, ECG, HFO	EMG and EEG	EMG	EEG and Local Field Potential (LFP)	EMG

The first chip presented in this table is DYNAP-SE, used to implement SNNs for the classification or detection of EMG (Donati et al., 2018, 2019; Ma et al., 2020a,b) and ECG (Bauer et al., 2019; Corradi et al., 2019) and to implement a simple spiking perceptron as part of a design to detect HFO in human intracranial EEG (Sharifshazileh et al., 2019). In particular, in Donati et al. (2018), Bauer et al. (2019), and Ma et al. (2020a,b) a spiking RNN is deployed for EMG/ECG signal separation to facilitate the classification with a linear read-out. SVM and linear least square approximation is used in the read out layer for Bauer et al. (2019) and Corradi et al. (2019) and overall accuracy of 91% and 95% for anomaly detection were reached, respectively. In Ma et al. (2020a) a RNN was implemented for discriminating three hand gesture using sEMG. Two hardware-friendly spike-based read-out models were used to evaluate the network performances: a rate-based state distance model, and a STDP model. The results show classification accuracy of the state distance method above 75%, better than the SVM approach, whereas the STDP learning rule only achieved 60% accuracy. The system was further expanded in Ma et al. (2020b), where an adapting spike conversion was introduced, improving the performances to 85%. In Donati et al. (2018), the state property of the spiking RNN on EMG was investigated for different hand-gestures. In Donati et al. (2019) the performance of a feedforward SNN and a hardware-friendly spike-based learning algorithm was investigated for hand-gesture recognition using superficial EMG and compared to traditional machine learning approaches, such as SVM. Results show that applying SVM and the spiking learning method on the spiking output of the hidden layer achieved a classification rate of 84% and 74%, respectively. Nevertheless, the latter show a power consumption of about 0.05*mW*, two orders of magnitude more power-efficient than the state-of-the-art embedded system (Benatti et al., 2015; Montagna et al., 2018).

Recently, the hand-gesture classification benchmark was implemented and compared on two digital neuromorphic platforms, i.e., Loihi (Davies et al., 2018) and ODIN/MorphIC (Frenkel et al., 2019a,b) and an embedded GPU, Nvidia Jetson Nano. The systems were using two different sensor modalities, event-driven sensors and EMG to perform sensor fusion. In particular, for processing vision inputs, a spiking Convolutional Neural Network (CNN) was implemented on Loihi and a spiking Multilayer Perceptron (MLP) was implemented on ODIN/MorphIC (Ceolini et al., 2020) while both the platforms used MLP for EMG processing. The difference in the two pipelines is due to the design properties of the neuromorphic systems (i.e., number of neurons, fan-in). However, in both cases, the fusion was performed on the layer before the one of classification, combining the output from the spiking CNN and the spiking MLP for Loihi, and from the two spiking MLPs on ODIN/MorphIC hardware. The same structure was implemented on the embedded GPU and the comparison was performed in terms of accuracy, power consumption, and latency showing that the neuromorphic chips are able to achieve the same accuracy with significantly smaller energy-delay product, 30× and 600× more efficient for Loihi and ODIN/MorphIC, respectively (Ceolini et al., 2020). The comparison was further extended in Azghadi et al. (2020), where the same task was applied to Field Programmable Gate Array (FPGA) and memristive implementations. Results show that neuromorphic hardware presents approximately two orders of magnitude improvement in the energy-delay product when compared to their FPGA counterparts, which highlights the prospective use of such architectures in edge computing.

### 4.3. Adaptation in Neuromorphic Processor

Local adaptation is an important aspect in extreme edge computing, specially for wearable devices. The current methods for training networks for biomedical signals rely on large datasets collected from different patients. However, when it comes to biological data, there is no “one size fits all.” Each patient and person has their own unique biological signature. Therefore, the field of Personalized Medicine (PM) has gained lots of attention in the past few years and the online on-edge adaptation feature of neuromorphic chips can be a game changer for PM.

As was discussed in section 3.1, there is on-going effort in designing spike-based online learning algorithms which can be implemented on neuromorphic chips.

Example of today's state of the art for on-chip learning are Intel's Loihi (Davies et al., 2018), DynapSEL and ROLLS chip from UZH/ETHZ (Qiao et al., 2015; Qiao and Indiveri, 2016), BrainScales from Heidelberg (Schemmel et al., 2010) and ODIN from UC Louvain (Frenkel et al., 2019a). Intel's Loihi includes a learning engine which can implement different learning rules, such as simple pairwise STDP, triplet STDP, reinforcement learning with synaptic tag assignments or any three factor learning rule implementation. DynapSEL, ROLLS and ODIN encompass the SDSP, also known as the Fusi learning rule, which is a form of semi-supervised learning rule that can support both unsupervised clustering applications and supervised learning with labels for shallow networks (Brader et al., 2007). Brainscales chip implements the STDP rule. Moreover, Spinnaker 1 and 2 (Furber et al., 2013; Mayr et al., 2019) can implement a wide variety of on-chip learning algorithms since their designs make use of ARM microcontrollers providing lots of configurability for the users. Table 2 summarizes the learning algorithms implemented on the neuromorphic chips that have been used for biomedical signal processing. Synaptic bit precision is an important parameter for online learning which is limited on chip due to the memory footprint.

### 4.4. Open Challenges

#### 4.4.1. System Integration

One of the main challenge in developing a device for Edge Computing is the integration of the sensors with the processor, which is generally valid, but even more in neuromorphic systems. In heterogeneous systems, where sensor and processor are not integrated in the same substrate, the main challenge is due to the lack of a standard in the protocol of communication. Although most of neuromorphic systems, both sensors and processors, implement Address-Event Representation (AER) protocol, they present slightly different implementations, i.e., parallel, serial, different AER address width, which makes the integration difficult. Another approach consists of designing sensors and processors on the same substrate. This solution is preferable for wearable solutions where edge computing is required, but it is currently not the case for any neuromorphic chips. Any neuromorphic system, in fact, comprises not only of the neuromorphic core but a digital infrastructure that surrounds the core, i.e., FPGAs and microcontrollers that allow the communication with the external world and the network configuration.

#### 4.4.2. Locality

The learning information for updating the weights of any on-chip network should be locally available to the synapse since otherwise this information should be “routed” to the synapse by wires which will take a significant amount of area on chip. The simplest form of learning which satisfies this requirement is Hebbian learning which has been implemented on a variety of neuromorphic chips in forms of unsupervised/semi-supervised learning (Schemmel et al., 2010; Qiao et al., 2015; Qiao and Indiveri, 2016; Frenkel et al., 2019a). However, Hebbian-based algorithms are limited in the tasks they can learn and to the best of our knowledge no large-scale task has been demonstrated using this rule. Since gradient descent-based algorithms, such as Backprop has had lots of success in deep learning, there are increasingly more spike-based error Backprop rules that are being developed as was discussed in section 3.1. These types of learning algorithms have recently been custom designed in the form of spike-based delta rule as back-bone of the Backprop algorithm. For example, single layer implementation of the delta rule has been designed in Payvand and Indiveri (2019) and employed for EMG classification (Donati et al., 2019). Expanding this to multi-layer networks involves non-local weight updates which limits its on-chip implementation. Making the Backprop algorithm local is a topic of on-going research which we have discussed in section 3.1.

#### 4.4.3. Weight Storage

The ideal weight storage for online on-chip learning should have the following properties: (i) non-volatility to keep the state of the learnt weights even when the power shuts down to reduce the time and energy footprints of reloading the weights to the chip. (ii) Linear update which allows the state of the memory to change linearly with the calculated update. (iii) Analog states which allows a full-precision for the weights. Non-volatile memristive devices have been proposed as a great potential for the weight storage and there is a large body of work combining the CMOS technology with that of the memristive devices to get the best of two worlds.

In the next section we provide a thorough review on the state of the art for the emerging memory devices and the efforts to integrate and use them in conjunction with neuromorphic chips.

## 5. Memristive Devices and Computing

The severe power and area constraints under which a neuromorphic processor for edge computing must work opened ways toward the investigation of beyond-CMOS solutions. Despite remaining in the early phase of its technological development, memristive devices have been drawing attention in the last decade thanks to their scalability, low-power operation, compatibility with CMOS chip power supply and CMOS fabrication process, and volatile/non-volatile properties. In section 5.1, we will introduce memristive devices and the properties that are appealing for adaptive extreme edge computing paradigms. In section 5.2, we will explore the role of memristive devices in neuromemristive systems and give examples of possible applications. In section 5.3, we will discuss the current challenges and the future perspectives of memristive technology.

### 5.1. Conventional and Wearable Memristive Devices

**Figure 4:**
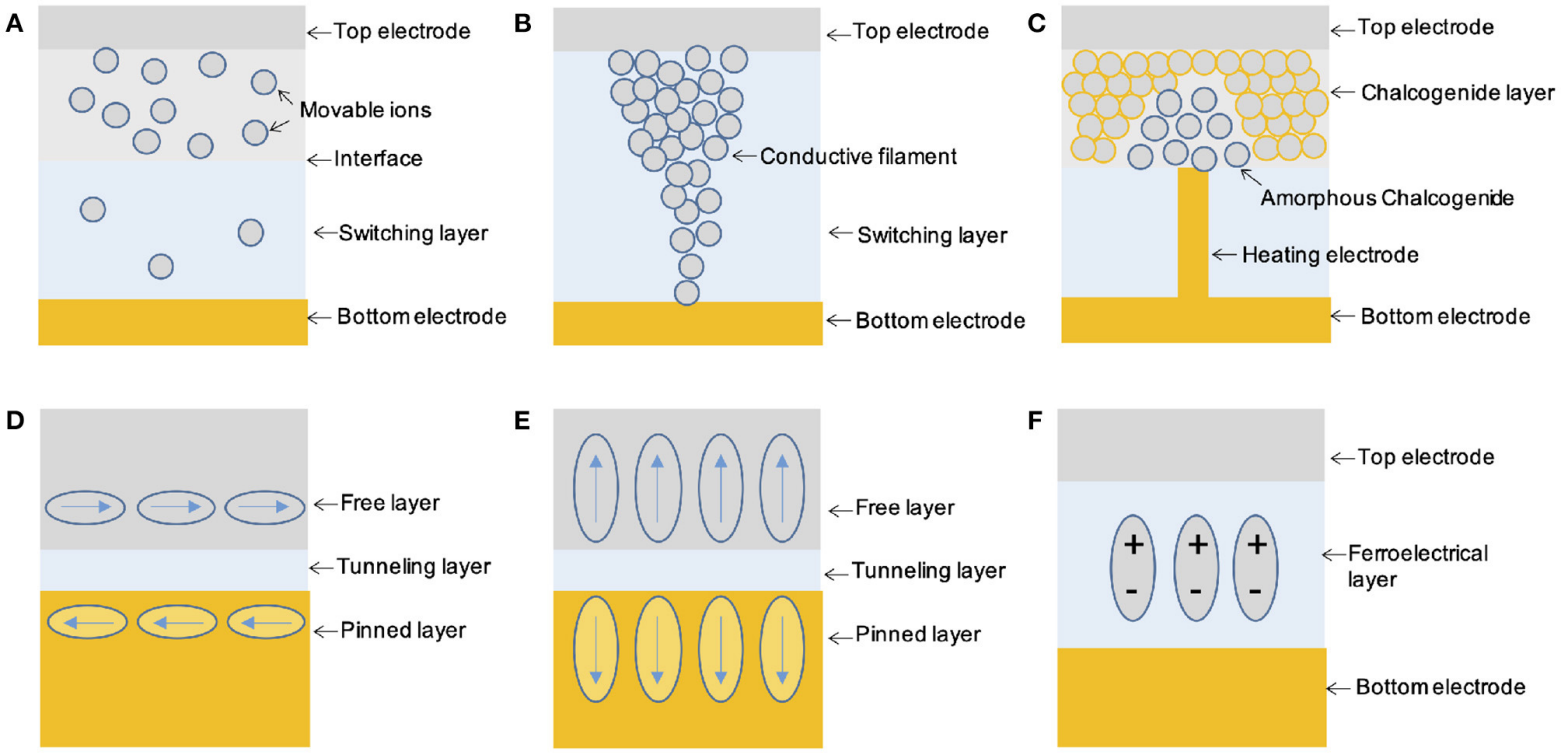
Memristive devices for neuromorphic computing. **(A)** Interface type RRAM device; **(B)** Filamentary RRAM device; **(C)** Phase change memory device; **(D)** MRAM device with in-plane spin polarization; **(E)** MRAM device with perpendicular spin polarization; **(F)** FTJ device.

Memristive devices, as the name suggested, are devices which can change and memorize their resistance states. They are usually two-terminal devices, however, can be implemented with various physical mechanisms, resulting in versatile existing forms, e.g., resistive random access memory (RRAM, Figures 4A,B) (Ielmini and Wong, 2018), phase change memory (PCM, Figure 4C) (Zhang et al., 2019), magnetic random access memory (MRAM, Figures 4D,E) (Miron et al., 2011), ferroelectric tunneling junction (FTJ, Figure 4F) (Wen et al., 2013), etc. The resistance memory of these devices can mimic the memory effect of the basic components of biological neural system, while the resistance changing can mimic the plasticity of biological synapse. Facilitated with their simplicity of two-terminal configuration and scalability to nanoscale, they are inherently suitable for the hardware implementation of brain-inspired computation materializing an artificial neural network, i.e., neuromorphic computation (Jo et al., 2010; Wang et al., 2016a).

This notation, in recent years, has incited wide investigations on the various memristive devices and on their applications in neural network learning and recognition, or, in short, memristive learning (Ohno et al., 2011; Kuzum et al., 2012; Alibart et al., 2013; Yang et al., 2013; Eryilmaz et al., 2014; Ambrogio et al., 2018). The memristive learning can enable energy efficient and low latency information process within a reduced size of systems abandoning the conventional von-Neumann architecture. Among other benefits, this will also make it possible to process information where they are acquired, i.e., within sensors, and reduce the bandwidth needed for transferring the sensor data to data center, accelerating the coming of the era of Internet-of-Things (IOT). Table 3 summarizes the key features of the main memristive device technologies for neuromorphic/wearable applications in terms of cell area, electrical characteristics, main advantages and challenges. It is worth noticing that some figures of merit in this context are radically different with respect to standard memory requirements. Indeed, while in the memory scenario higher read currents enable faster reading speed, in neuromorphic applications currents as low as possible are preferred, since the current is a limiting factor for neurons' fan-out. Similarly, SET and RESET times should be as fast as possible in memory applications, while in our applications this requirement can be relaxed thanks to the lower operating frequency of the neurons (20–100 Hz). Moreover, the number of achievable conductance levels has to be increased (Ielmini and Pedretti, 2020). Some non-idealities which are usually detrimental for memory applications, for instance, stochasticity of switching parameters, are even beneficial for the neural networks. It is also worth noticing that the figures of merits in Table 3 are the best results extracted from different devices. There are no devices that simultaneously show all these best merits. For instance, if the RRAM and PCM devices are engineered to have multilevel states for multilevel synaptic application, lower endurance would be expected. However, in another aspect, devices with only binary states can also be used with dedicated binarized neural networks and stochastic algorithms.

**Table 3:** Key features of non-volatile memristive devices.

	**RRAM**	**PCM**	**MRAM**	**FTJ**
Cell area [min. feature size]	4*F*^2^ (IRDS, 2020)	4*F*^2^ (IRDS, 2020)	9*F*^2^ (Rho et al., 2017)	4*F*^2^ (IRDS, 2020)
Retention	>10 years (Goux et al., 2014)	>10 years (Cheng et al., 2012)	>10 years (Golonzka et al., 2018)	>10 years (Udayakumar et al., 2013)
Endurance	10^12^ (Kim et al., 2011; Lee et al., 2011)	10^11^ (Kim et al., 2010)	10^12^ (Saida et al., 2017)	>10^15^ (Udayakumar et al., 2013)
SET/RESET time	100 ps (Torrezan et al., 2011)	>100 ns, 10 ns	20 ns (Jan et al., 2018)	30 ns, 30 ns
	85 ps (Choi et al., 2016)	(IRDS, 2020)	3 ns (Kitagawa et al., 2012)	(Francois et al., 2019)
Read current	100 pA (Luo et al., 2016)	25 μA (De Sandre et al., 2010)	20 μA (Kitagawa et al., 2012)	0.8 nA (Bruno et al., 2016), device diameter 300 nm)
Write energy per bit	20 fJ (Kang et al., 2015)	~100 fJ (Xiong et al., 2011)	90 fJ (Kitagawa et al., 2012)	<10 fJ (Francois et al., 2019)
Main features	Scalability, multilevel, speed, low energy	Scalability, multilevel, low voltage	Endurance, low power	Endurance, low power, speed
Challenges	Variability	RESET current, temperature stability, resistance drift	Density, scalability, variability	Scalability

In addition to the commonly referred non-volatile type of memristive switching, the RRAM device can also show volatile behavior, which usually occurs when active materials, such as silver or copper are used as electrode. The relatively long retention time of the volatile behavior [tens of milliseconds to seconds (Covi et al., 2019)] is then found to be similar to the timescale of short term memory, and naturally was proposed to mimic the short term memory effect of biological synapses (Wang et al., 2017, 2019a). Practical examples where volatile devices can be useful are voice (Zhong et al., 2021) and spatiotemporal (Wang et al., 2021) recognition. In the latter case, thanks to device volatility, the network does not need any training and is naturally configured to detect events which occur in time (Du et al., 2017; Wang et al., 2018c, 2019a; Moon et al., 2019). Moreover, it should be mentioned that volatile devices have also shown potential when used as reservoir in a computing system for temporal information processing and time-series prediction, and solver of second-order non-linear dynamic tasks (Du et al., 2017; Moon et al., 2019).

Although most researches on memristive devices are carried on rigid silicon substrates, the simple structure of memristive devices can also be realized on flexible substrates (Shi et al., 2020), which opens new interesting possibilities for realizing local computation within wearable devices (Shang et al., 2017; Dang et al., 2019).

The conventional floating gate non-volatile memories could also be used for synaptic and neuromorphic application. For instance, Malavena et al. (2019) show that floating gate memories in NOR Flash array can be used for pattern learning via STDP weight update algorithms. Floating gate transistors can also be fabricated in two-terminal configuration, which can behave like a memristive device and be used for various neuromorphic applications (Danial et al., 2019). The mature fabrication process and increasing integration capability of floating gate transistors pose great advantages over emerging non-volatile memories.

### 5.2. Memristive Devices for Neuromorphic Computing

#### 5.2.1. Memristive Neural Components

As mentioned in section 5.1, the primary function of memristive devices is the usage as synaptic devices to implement the memory and plasticity of biological synapses. However, there are increasing interests for these devices to be utilized to implement nanoscale artificial neurons.

On the neuron side, the memristive device gradual internal state change and its consequently abrupt switching closely mimic the integrate-and-fire behavior of biological neurons (Mehonic and Kenyon, 2016; Tuma et al., 2016; Suresh et al., 2019, Figures 5A–C). Due to the sample structure and nanometer level scalability, memristive neurons can be much more compact than current CMOS neurons which might consist of current sensor, ADC, Digital to Analog Converter (DAC), and capacitors, all of which are expensive to implement in current CMOS technology in terms of area and/or power consumption (Kwon et al., 2018). The implementation of memristive neurons will also enable full memristive neuromorphic computing (Wang et al., 2018c), which promises further increases in the integration of the hardware neuromorphic computing.

**Figure 5:**
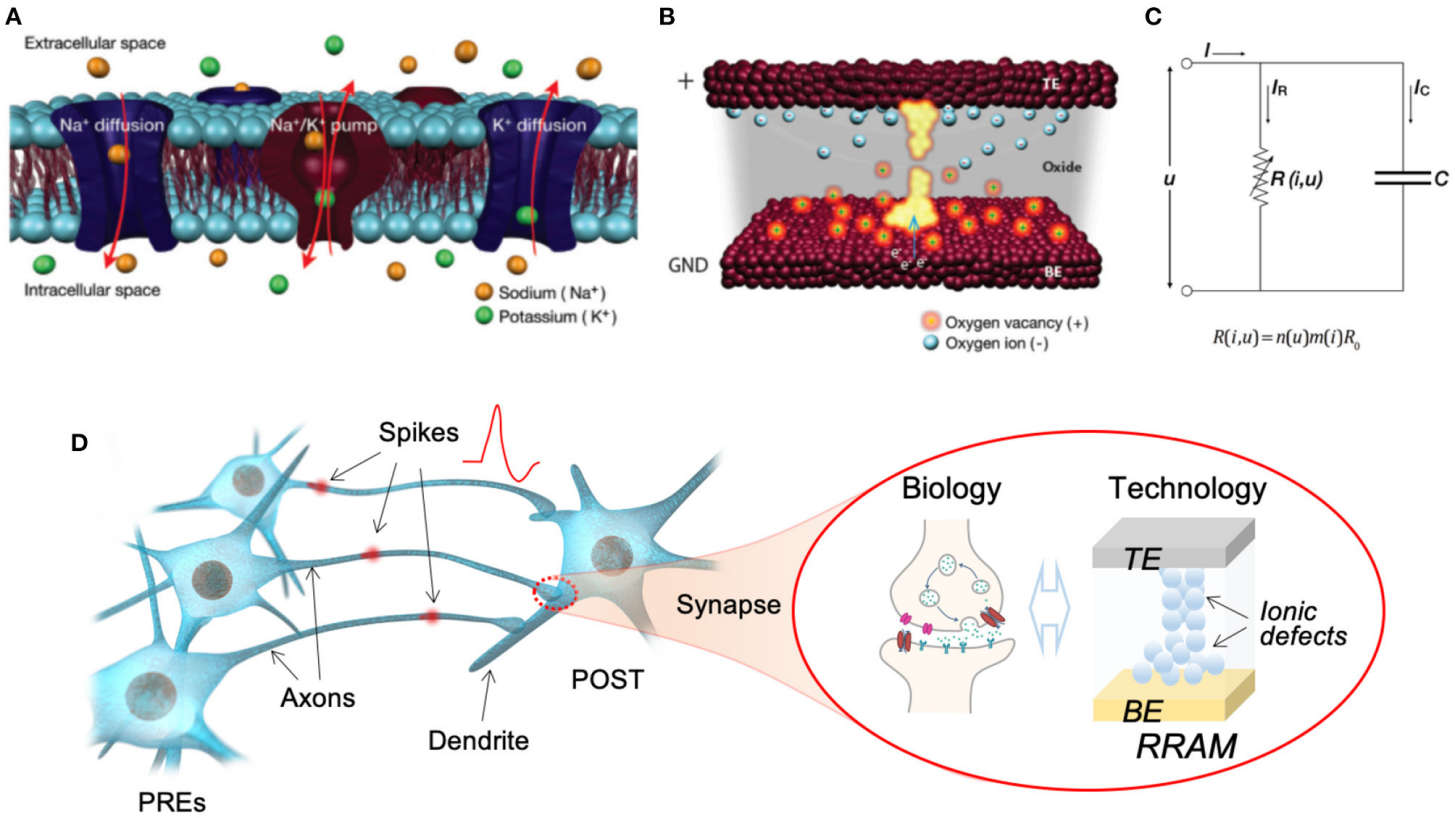
Memristive devices as synapse or neuron for neuromorphic computing. **(A–C)** Memristive device act as threshold device for the firing function of biological neuron (Mehonic and Kenyon, 2016), reproduced under the CC BY license. **(D)** Conceptual illustration of memristive device as artificial synapse for brain-like neuromorphic computing (Wang et al., 2018b), reproduced under the CC BY-NC license.

On the synaptic side, the key feature of the biological synapses is their plasticity, i.e., tunable weight, which can be generally implemented by resistance or conductance modification in the memristive devices (Figure 5D). Fundamental learning rules based on STDP have already been widely explored (Kuzum et al., 2012; Wang et al., 2015; Covi et al., 2016, 2018; Mulaosmanovic et al., 2017). Spatial spiking pattern recognition (Pedretti et al., 2017), spiking co-incidence detection (Sebastian et al., 2017; Prezioso et al., 2018), and spatial-temporal correlation (Wang et al., 2018b, 2019b) has been reported recently. Synaptic metaplasticity, such as paired-pulse facilitation, can also be achieved via various device operation mechanism (Wang et al., 2017; Zhu et al., 2017b; Wu et al., 2018).

#### 5.2.2. Memristive Neural Networks

There are generally two approaches for a hardware neuromorphic system utilizing memristive devices as synapses: (i) deep learning accelerator, accelerating the artificial neural network computing with multiple layer and error back-propagation, as well as it's variations, like convolutional neural network, recurrent neural network, etc.; (ii) brain-like computing, attempting to closely mimicking the behaviors of biological neural system, like spike representation (Figure 5D) and collective decision making behavior. In the deep learning accelerator approach, on-line training places more requirements for the memristive synapses. For instance, linear and symmetrical weight update is crucial for the on-line training (Burr et al., 2015; Ambrogio et al., 2018), while off-line training ignores it since the synaptic weight can be programmed to the memristive device with fine tuning and iterative verify (Yao et al., 2020). In deep learning, therefore, the minimization of device variability becomes of utmost importance to enable online training, as already proposed in some works (Shafiee et al., 2016; Cheng et al., 2017; Song et al., 2017; Imani et al., 2019; Ankit et al., 2020).

Collective decision making is an important feature of the brain computing, which requires high parallelism and, consequently, low current devices. For instance, this feature is the essential for Hopfield neural network (Hopfield, 1982), cellular neural network (Duan et al., 2015), and coupled oscillators (Romera et al., 2018). In the Hopfield neural network, the system automatically evolves to its energy minimization points leading the functionality of associative memory. The use of Hopfield like recurrent neural networks (RNNs) with memristive devices has already been successfully demonstrated in a variety of tasks (Milo et al., 2017; Wang et al., 2020b). As an example of memristive based coupled oscillator network, Ignatov et al. (2017) used a network of self-sustained van der Pol oscillators coupled with oxide-based memristive devices to investigate the temporal binding problem, which is a well-known issue in the field of cognitive neuroscience. In this experiment, the network is able to emulate an optical illusion which shows two patterns depending on the influence of attention. This means that the network is able to select relevant information from a pool of inputs, as in the case of a system collecting signals from multiple sensors.

#### 5.2.3. Applications of Memristive Neural Networks

At present, Backprop has already exploited for offline training of moderate size memristive neural networks (Valentian et al., 2019). Backpropagation based on online training schemes has also been implemented in several memristive deep learning accelerators (Li et al., 2018a; Wang et al., 2019d; Yao et al., 2020), showing great success of memristive array on accelerating the deep learning training and adaptive to some device non-ideal characteristics. The readers can refer to more comprehensive review papers for more details (Wang et al., 2020a; Zhang et al., 2020; Berggren et al., 2021). In these works, however, the error backpropagation—a backward vector matrix multiplication, and the gradient descent calculation—a vector-vector out-product, are both conducted in hosting computer. The implementation of these two operations in memristive array will further improve the performance of the deep learning accelerators, while Hebbian-based learning algorithms could potentially bypass these operations.

Online versions of Backprop, as discussed in section 3, are very recent and a memristive-based hardware demonstration is not yet available, despite some work in this direction is being done (Payvand et al., 2020b). To implement adaptation, biologically plausible algorithms able to cope with the non-ideal characteristics of memristive devices are needed. Hebbian-based algorithms are expected to fulfill all these requirements. However, memristive technology with Hebbian-based learning algorithms has been so far mainly used in relatively simple networks. More recently, systems able of solving different tasks, such as speech recognition (Park et al., 2015), and exploring different architectures and learning algorithms are being investigated. In particular, the benefits of exploiting sparsity, mentioned in section 3.2, are demonstrated for feature extraction and image classification in networks trained with stochastic gradient descend and winner-take-all learning algorithms (Sheridan et al., 2016), as well as in hierarchical temporal memory, which does not need training (Krestinskaya and James, 2018).

In the latest years, memristive devices have been used in applications closer to biology, enabling hybrid biological-artificial systems (Serb et al., 2020) and investigating biomedical applications, ranging from speech and emotion recognition (Saleh et al., 2015) to biosignal (Kudithipudi et al., 2016) and medical image (Zhu et al., 2017a) processing. An interesting application is the one of memristive biosensors, which Tzouvadaki et al. (2018) used to implement a system for cancer diagnostic. The innovative use of memristive properties was demonstrated in hardware and opens the way to a broader use of memristive technology where sensors and computing co-exist in the same system or, possibly, in the same device. Finally, a recent work utilizes memristor array for neural signal processing which shows three-orders-of-magnitude improvements in power efficiency compared with literature of CMOS ASIC technology (Liu et al., 2020).

### 5.3. Open Challenges and Future Work

#### 5.3.1. Device Non-idealities

Implementation of mainstream deep learning algorithms with Backprop learning rule and memristive synapses imposes some requirements for the memristive device, including linear current-voltage relation for reading, analog conductance tuning, linear and symmetric weight update, long retention time, high endurance, etc. (Gokmen and Vlasov, 2016). However, no single device can fulfill all these requirements simultaneously.

Various techniques have been proposed to compensate the device non-idealities. For instance, to compensate the non-linear current-voltage relation for reading, fixed read voltage with variable pulse width or pulse number can be used for synaptic weight reading, and the readout is represented by the charge accumulation in the output nodes (Cai et al., 2019). Linear and symmetric weight update is crucial for accurate online learning of a memristive multilayer neural network with Backprop learning rule (Burr et al., 2015). However, PCM devices usually only show gradual switching in set direction (weight potentiation), while RRAM devices show gradual switching in reset direction (weight depression). To achieve linear and symmetric weight update, differential pair with two of these devices are usually used. For a differential pair with two PCM devices, the potentiation is achieved by applying set pulses on the positive part and the depression is achieved by applying set pulses on the negative part, thus gradual weight update in both potentiation and depression can be achieved. To further enhance the linearity of weight update, a minor conductance pair consisting of capacitors can be used for frequent but smaller weight update, and finally transferred to the major pair periodically (Ambrogio et al., 2018). Another option to improve device linearity is limiting the device dynamic range in a region far from saturation and where the weight update is linear (Wang et al., 2016b; Woo et al., 2016).

In addition to mitigate the non-idealities of memristive devices, more and more research efforts are made to exploit these non-idealities for brain-like computations. For instance, the stochasticity or noise in reading of memristive device can be used for the probability computation for restricted Boltzmann machine (Mahmoodi et al., 2019), or escape for local minimization points in a Hopfield neural network (Cai et al., 2020). The Ag filament based resistive switching device shows short retention time and high switching dynamics, thus was proposed for reservoir computing (Midya et al., 2019) and spatiotemporal computing (Wang et al., 2019a) to process time-encoded information.

#### 5.3.2. Co-integration of Hybrid CMOS-Memristive Neuromorphic Systems

The main steps to be taken to exploit the full potential of an ASIC for end-to-end processing system go through the integration of memristive devices and sensors with CMOS technology. Indeed, the works presented so far are based either on simulations or on real device data, or on memristive chips interfaced with some standard digital hardware. Despite integration of CMOS technology has been demonstrated for non-volatile resistive switching devices already at a commercial level (Yang-Scharlotta et al., 2014; Hayakawa et al., 2015), the design of co-integrated memristive-based neuromorphic processors is still under development. We envisage a three-phase process to achieve a fully integrated system.

The first one is the co-integration of non-volatile memristive devices with some peripheral circuits (Hirtzlin et al., 2020) and to implement some logic and multiply-and-accumulate (MAC) operations (Chen et al., 2019), which reaches the maturity with the demonstration of a fully co-integrated SNN with analog neurons and memristive synapses (Valentian et al., 2019). The second phase is the co-integration of different technologies. Despite this approach results in higher fabrication costs, it presents several advantages in terms of system performance, which can be more compact and potentially more power efficient. In particular, the co-integration of non-volatile and volatile memristive devices can lead to a fully memristive approach. As an example, Wang et al. (2018c) exploit volatile memristive devices to emulate stochastic neurons and non-volatile memristive devices to store the synaptic weights on the same chip, thus demonstrating the feasibility and the advantages of the dual technology co-integration process. Eventually, the final step which has to be taken in the development of a dedicated ASIC for wearable edge computing is the co-integration of sensors and memristive-based systems. Shulaker et al. (2017) tackled this challenge by designing and fabricating a gas sensing system able of gas classification. The system uses RRAM arrays as memory, Carbon Nanotube Field Effect Transistor (CNFET) for computation and gas sensing, both 3D monolithically integrated on CMOS circuits, which carry out computation and allow memory access.

Finally, there are some further aspects to be considered in order to ensure a successful co-integration. At advanced technological nodes, the power supply of the chip might be lower than the voltages required to operate memristive devices, especially when a forming operation is required. To avoid the use of charge pump circuits, as it is necessary in Flash technology, a possible solution is investigating forming-free devices (Hansen et al., 2018) and low-voltage operation devices with programming voltages <1 V (Gilbert et al., 2013; Guo et al., 2020).

#### 5.3.3. Learning With Memristive Devices

Adaptability is a feature of paramount importance in smart wearable devices, which need to be able to learn the unique feature of their user. This calls for the implementation of lifelong learning paradigms, i.e., the ability of continuously learning new features from experience. Typically, a network has a limited memory capacity dependent on the network size and architecture. Once the maximum number of experiences is recorded, new features learned will erase old ones, thus originating the phenomenon of catastrophic forgetting.

The problem of an efficient implementation of continual learning has been thoroughly investigated (Parisi et al., 2019). In the current scenario, a dichotomy exist between backprop-based ANNs, which have very high accuracy but a limited memory capacity, and brain-inspired SNNs, which feature higher memory capacity thanks to their higher flexibility, but at the cost of lower accuracy. Models used to reduce the effect of forgetting stability-plasticity problem are described in section 3.3. The use of memristive devices in such networks is still an open point. It is possible that memristive device will be beneficial to increase the network capacity (Brivio et al., 2018) at no extra computational cost thanks to their slow approach to the boundaries (Frascaroli et al., 2018), but so far this topic is still quite unexplored. An interesting approach is proposed by Muñoz-Martín et al. (2019), where the key strengths of supervised convolutional ANNs, unsupervised SNNs, and memristive devices are combined in a single system. The results indicate that this approach is robust against catastrophic forgetting, whilst reaching 93% accuracy when tested with both trained and non-trained classes.

## 6. Discussion and Conclusions

In this study, we presented the state-of-the-art core elements that enable the development of wearable devices for healthcare and biomedical applications with extreme edge adaptive computing capability. Various sensors that can collect different bio-signals from the human body are investigated. There is a variety of sensing specifications in terms of size, resolution, mechanical flexibility and output signals that needs to be considered along with their analog readout circuit at a limited amount of power consumption. However, when the real-time processing of these signals is deployed on edge, severe constraints raise in terms of power efficiency, fast response times, and accuracy in the data classification. The widely-used solution is to find a trade-off between the energy and computational capacity, or send the data to the cloud. However, these strategies are not ideal and slow down the development of wearable smart sensing. Another important aspect to be considered is the matching of the time constants with the intended application. Indeed, electronic systems are intrinsically much faster than real-time events. This property can be exploited to carry out accelerated-time simulations, which are extremely appealing to investigate processes occurring in very long time scales (Schemmel et al., 2020). In systems interacting with the environment, instead, the time constants should be slowed down to match real-time ones in order to optimize energy utilization and enable a seamless processing of biological signals. To meet all the requirements, the development of a platform needs to be optimized in synergy with the other elements and every aspect of the design, from the learning algorithms to the architecture.

Continual learning is required for adaptive wearable devices. In this respect, brain-inspired algorithms promise to be valid alternatives to standard machine learning approaches, such as Backprop and BPTT. The exploitation of sparsity in network connectivity increases the power efficiency by optimizing the use of the available memory. However, the problem of algorithmic robustness to non-ideal hardware (such as noise and variability) and the problems of forgetting and information transfer between tasks still persist and have to be solved in combination with neuromorphic and emerging technologies. SNNs are conceptually ideal for low-power in-memory computing. Their event-based approach, which exploits the low latency of electronics to route the spikes to the correct neuron (Moradi et al., 2017), together with the use of analog subthreshold circuits to reproduce biological timescales, allows fast response times of the network while enabling smooth real-time processing of data. The encoding of the incoming signals into spikes is however still challenging. Moreover, a fully CMOS-based approach has two major technological issues. First, capacitors used to implement biological time constants are massive and may consume up to 60% of the chip area. Memristive technology can be beneficial in this respect, as volatile devices offer a compact alternative to CMOS capacitors. Second, the network configuration and the synaptic weights are usually stored in Ternary Content-Addressable Memory (TCAM)s and in SRAMs, respectively, which hold the state only in the presence of a power supply. This implies that (i) power supply cannot be switched off during normal system operation unless the relevant information is first stored somewhere else and (ii) at every start up of the system, the information on the network has to be uploaded, which may take tens of minutes. Non-volatile memristive device-based versions of TCAM dramatically reduce the initialization times, since the information is already stored in the network. Moreover, memristive-based synapses can also enable normally-off computing paradigms, thus further improving power efficiency.

Besides low-power operation in a small footprint, memristive devices also offer noisy properties, which—if exploited in the right way—might facilitate the implementation of stochastic learning algorithms. However, the technology is still at its infancy and fabrication processes are still under development, yielding high device variability, which makes it difficult to produce reliable multi-bit memory.

The focus of this study is describing the technological challenges and possible solutions to bring computing abilities on the edge. However, there are other practical aspects that may pose a hurdle for the deployment of the envisaged high performance edge biomedical systems (Figure 1). (i) *Data set*. The available biomedical data sets may not represent uniformly the human population, since they are mainly collected in countries with a granted basic healthcare system. In this context, online adaptation enables the biomedical device to learn directly from the signal of the user, which should mitigate data set related issues. (ii) *Need for interpretability*. Especially in high-risk scenarios, such as in medicine, where a false positive or negative can have a huge impact on the patient, having transparent Artificial Intelligence (AI) models and systems is of paramount importance to support medical doctors in a decision (Barredo Arrieta et al., 2020). (iii) *Legal responsibility*. Machine Learning (ML) is not unerring. When it does, for example in automotive or healthcare scenarios, our current legal systems lack laws that can clearly define responsibilities (Eshraghian, 2020). (iv) *Generalization performance*. Human intelligence outperforms AI when dealing with generalization tasks, even though some efforts are already devoted to improving this aspect (McKinney et al., 2020). If successful, AI can provide a valid instrument for medical doctors for an early detection of a pathological abnormality (even before the patient displays symptoms), an early start of appropriate therapy, and an overall improvement of prognosis. However, these aspects lie well-beyond the scope of this study and deserve an extensive review on their own.

In summary, the ultimate goal toward smart wearable sensing with edge computing capabilities relies on a bespoke platform embedding sensors, front-end circuit interface, neuromorphic processor and memristive devices. This platform requires high-compatibility of existing sensing technologies with CMOS circuitry and memristive devices to move the intelligent algorithm into the wearable edge without significantly increase the cost in energy. New solutions are needed to enhance the performance of local adaptive learning rules to be competitive with the accuracy of Backprop. Novel encoding techniques to allow seamless communication from sensors to neuromorphic chips have to be developed and flanked by efficient event-based algorithms. So far there is not a uniquely ideal solution, but we envisage that a holistic approach where all the elements of the system are co-designed as a whole is the key to build low-power end-to-end real-time adaptive systems for next-generation smart wearable devices.

## Author Contributions

XL and HH: wearable sensors. DK: biologically plausible algorithms. MP and ED: signal processing and neuromorphic computing. EC and WW: memristive devices. EC: led and coordinated the cooperative writing and all discussions. All authors equally contributed to the manuscript, actively participating to the discussions and to the writing.

## Conflict of Interest

The authors declare that the research was conducted in the absence of any commercial or financial relationships that could be construed as a potential conflict of interest. The reviewer JE declared a past co-authorship with one of the authors MP to the handling editor.
